# Data‐Driven Materials Science: Status, Challenges, and Perspectives

**DOI:** 10.1002/advs.201903667

**Published:** 2020-01-22

**Authors:** Lauri Himanen, Amber Geurts, Adam Stuart Foster, Patrick Rinke

*Adv. Sci.* **2019**, *6*, 1900808

In the originally published article, **Table** **1** and **Table** **2** did not contain timely data. The corrected entries of Table 1 and Table 2 are presented below.

To correct these errors, two new entries have been added: QCArchive^1^ and NanoMine.^2^, ^3^ Also, the entry for MARVEL NCCR has been renamed to Materials Cloud and corrected in Table 1 and Table 2; and the entries Open Quantum Materials Database (OQMD), SUNCAT, MaterialsZone, and Exabyte have been corrected in Table 2. Finally, the description for data upload and web API have been clarified in the caption of Table 2. The authors apologize for any misunderstanding these errors may have caused.

**Corrections to Table 1:** List of current major materials data non‐commercial infrastructures. Note that some platforms are named after the leading research project and may host multiple services under different names. As contact person we listed the director(s) of each infrastructure, in such cases, where they were clearly identifiable. Data volume numbers reflect the state in April 2019

Name	Website	Contact	Overview	Ref.
QCArchive	qcarchive.molssi.org	Daniel Smith, MolSSI	A central source to compile, aggregate, query, and share quantum chemistry data. Access to millions of computational molecular science results.	1
NanoMine	materialsmine.org	Catherine Brinson, Duke University	Material informatics platform for polymer nanocomposites. Provides tools for curating, visualizing, pre‐processing and analyzing nanocomposite data.	2, 3
Materials Cloud	materialscloud.org	Nicola Marzari, EPFL	Materials informatics platform for data‐driven high‐throughput quantum simulations, powered by the AiiDA‐infrastructure (aiida.net).	4

**Corrections to Table 2:** Services provided by the selected materials data infrastructures. Open Access: provides partial or full free access to data. Computational data: contains data originating from software simulations. Experimental data: contains data originating from experiments. Data upload: Allows upload of external data, services that support issuing Digital Object Identifiers have been marked with (DOI). Workflow management tools: provides or collaborates in the development of open‐source software tools for workflow management. Web API: provides an interface for accessing data remotely with automated scripts. Data analysis tools: provides online or offline data analysis tools, including machine learning

	Open Access	Comp. data	Exp. data	Data upload (DOIs)	Workflow management tools	Web API	Data analysis tools
Materials Cloud	√	√		√(DOI)	√	√	√
NanoMine	√		√	√			√
Open Quantum Materials Database	√	√				√	√
SUNCAT	√	√		√		√	√
QCArchive	√	√		√	√	√	√
Exabyte.io	√^a)^	√		√		√	√
MaterialsZone			√	√			√

^a)^ Open Access to a subset of data.
